# Antioxidant and antimicrobial properties of Pelargonium sidoides DC and lactoferrin combination

**DOI:** 10.1042/BSR20203284

**Published:** 2020-11-13

**Authors:** Michela Terlizzi, Chiara Colarusso, Umberto Di Maio, Antonino Bagnulo, Aldo Pinto, Rosalinda Sorrentino

**Affiliations:** 1 Department of Pharmacy, University of Salerno, Fisciano, SA, Italy; 2 ImmunePharma srl, Fisciano, SA, Italy; 3 Shedir Pharma srl, Piano di Sorrento, Italy; 4 Neilos srl, Piano di Sorrento, Italy

**Keywords:** anti-inflammatory, anti-microbial activity, anti-oxidant, Lactoferrin, Pelargonium sidoides DC

## Abstract

Lactoferrin (LAT), a multifunctional protein involved in numerous physiological functions, and the medicinal plant Pelargonium sidoides DC (PEL) have been described for their anti-inflammatory properties. Because the main advantage of natural products consists in administering them in combination rather than as single compound, we aimed to understand whether the combination of PEL and LAT, herein PELIRGOSTIM, could still prove beneficial or additive/synergistic activities during inflammatory conditions. To pursue this goal, we used macrophagic cells (J774.1) and treated them with PEL and LAT in a concentration-dependent manner. We found that PELIRGOSTIM was able to reduce the levels of reactive oxygen species (ROS) and nitrite, effects that were correlated to the release of lower levels of IL-1β after LPS treatment. In addition, the combination of PEL and LAT showed bacteriostatic activities against *Staphylococcus aureus* and *Escherichia coli* which had limited growth starting from 5 hours up to 20 hours. This effect was stronger than that observed for penicillin/streptomycin.

Our results provide PELIRGOSTIM as an innovative combination of natural products capable to prevent inflammation-, oxidative stress- and microbial-related disorders.

## Introduction

Pelargonium sidoides DC (PEL) (Geraniaceae) is an African medicinal plant, traditionally used for curing various ailments, including diarrhoea, colic, gastritis, tuberculosis, cough, hepatic disorders, menstrual complaints and gonorrhoea [1]. The common name, *umckaloabo*, represents the Zulu word describing ´severe cough’. Indeed, its extracts are successfully employed in modern phytotherapy in Europe to cure infectious diseases of the respiratory tract [1].

Inspired by the healing of his tuberculosis, Charles Henry Stevens introduced this phytomedical drug to England in 1897 [2], and since then, the use of this plant has increased more and more interest because of its beneficial properties. Pharmacological studies have demonstrated the usefulness of PEL in the treatment of several disorders due to its anti-inflammatory, antioxidant and immunomodulatory activities [3] and its antiproliferative effect [4]. In our very recent paper, we demonstrated that PEL together with other natural products can exert antimicrobial activities [5]. So far, many studies have been conducted on the combination of PEL with other compounds, in order to identify new synergistic or enhanced pharmacological effects. In the present study, we have investigated the anti-inflammatory, antioxidant and antimicrobial activity of Pelargonium sidoides DC (PEL) combined with lactoferrin (LAT), a multifunctional protein that participates to numerous physiological functions.

LAT is a nonhemic iron-binding glycoprotein of the transferrin family [6]. It is present in many biological fluids and tissues, particularly in neutrophil granules [6]. It is involved in iron transportation, immune responses, organ morphogenesis and promotes wound healing and bone growth [7]. It has also been described as beneficial to prevent cancer [7] and to exert beneficial cardiovascular properties such as lipid reduction, antihypertensive and antithrombotic effects [8]. Moreover, its iron chelating ability underlies its anti-inflammatory, antioxidant and antimicrobial properties [9].

Therefore, based on the literature, the main goal of the present study was to evaluate whether PEL and LAT combination could be beneficial. We found that this novel combination, PEL+LAT, herein PELIRGOSTIM, had anti-inflammatory, antioxidant and antimicrobial activities, and that could be an alternative therapeutic strategy to the actual antimicrobial therapy.

## Materials and methods

### Sample preparation

Pelargonium sidoides DC (PEL; Cod: LIP00538), LAT and their combination (patented as PELIRGOSTIM® with number 102018000002457) were obtained from Shedir Pharma s.r.l. as dried powder, reconstituted with bi-distilled water at the stock concentration of 10 mg/ml. Specifically, the samples were tested, alone and in combination, as follows:

- Pelargonium sidoides DC (PEL; 0.01, 0.1, 1, 10 and 100 μg/ml)
- Lactoferrin (LAT; 0.01, 0.1, 1, 10, 100 μg/ml)
- PEL (10 μg/ml) + LAT (1 μg/ml)

The study was conducted for 9 months.

### MTT assay

To assess cell viability, 3-(4,5-dimethylthiazol-2-yl)-2,5-diphenyltetrazolium bromide (MTT) was added to medium-free cells post 24 h treatment. DMSO was used to dissolve the purple formazan crystals. The formazan concentration was determined by measuring the optical density. The data are presented as absorbance (550 nm) versus treatment.

### Cells cultures

J774.1 murine macrophage cells line were cultured in Dulbecco’s Modified Eagle Medium (DMEM) (Cambrex Biosciences, Microtech, Naples, Italy) supplemented with 10% foetal bovine serum (FBS), 100 units/ml penicillin, 100 units/ml streptomycin and 2 mM L-glutamine (Cambrex Biosciences, Microtech, Naples, Italy). Cells were seeded (5 × 10^4^ cells/well) 24 h before treatment. Then, cells were treated for 24 h with PEL (0.01, 0.1, 1, 10 and 100 μg/ml) and LAT (0.01, 0.1, 1, 10 and 100 μg/ml) ± LPS (0.1 µg/ml, Alexis, Vincibiochem, Italy). The following combinations have also been tested: PEL+LAT±LPS.

### Cytokine measurements

IL-1β was measured in cell-free supernatants obtained from the J774.1 cultures. Time-course experiments were preliminary performed to define the optimal release of IL-1β according to a positive control, LPS±ATP (0.5 mM). We found that J774.1 macrophage cell cultures released higher levels of IL-1β after 24 h of treatment. IL-1β was detected by means of a commercially available Enzyme-Linked Immunosorbent Assay Kits (ELISAs) (eBioscience, CA, U.S.A.; R&D Systems, U.S.A.). Cytokine levels were expressed as pg/ml.

### DCHF-DA assay

2′,7′-Dichlorodihydrofluorescein diacetate (DCHF-DA) is a cell-permeable probe used to detect intracellular reactive oxygen species (ROS). DCHF-DA probe is nonfluorescent in its initial form but it undergoes multistep conversion inside the cell that results in the formation of fluorescent product dichlorofluorescein (DCF), in presence of intracellular ROS. Briefly, J774.1 cells were plated (10^5^ cells/well) and treated as described above for 45 min [10]. After the subsequent incubation with DCHF-DA 10 μM for 15 min at 37°C, the flow-cytometry analysis was performed (BD FacsCalibur Milan, Italy). This time point was chosen according to the capability of LPS to induce oxidative stress [11]. Data were expressed as percentage of DCHF^+^ cells.

### Nitrite assay

J774.1 cells were plated (5 × 10^4^ cells/well) and treated as described above for 24 h. Nitrite levels in the culture supernatants were determined using the colorimetric Griess reaction (Sigma, St. Louis, MO, U.S.A.). Absorbance was measured with a plate reader at 550 nm. The concentration of nitrite (NO_2_^−^) was determined from standard curves constructed with serial concentrations of sodium nitrite (NaNO_2_).

### Bacterial cultures

*Staphylococcus aureus* (ATCC-6538; 0.5 × 10^6^ CFU/ml) and *Escherichia coli* (ATCC-25922; 0.5 × 10^6^ CFU/ml) were cultured in Lysogeny Broth (LB) and treated as follow: LB (Negative Control), Penicillin/Streptomycin (Pen/Strep) 5× (5%) (Positive Control), PEL 10 μg/ml, LAT 1 μg/ml, PEL+LAT. These concentrations were chosen upon preliminary minimum inhibitory concentration (MIC) studies. The bacterial growth was evaluated measuring absorbance at 595 nm, at the following experimental times: 0, 1, 2, 3, 4, 5, 6, 7 and 20 h.

### Statistical analysis

Data are reported as scatter dot plots, showing the mean ± SEM. Statistical differences were assessed with one-way ANOVA followed by Bonferroni’s post-test, or two-way ANOVA followed by Tukey’s multiple comparison post-test, as appropriate. *P* values less than 0.05 were considered as significant.

The statistical analysis was performed by using GraphPad prism 8.4.3 version (San Diego, U.S.A.).

## Results

### PEL and LAT combination showed anti-inflammatory activity

**Figure 1:**
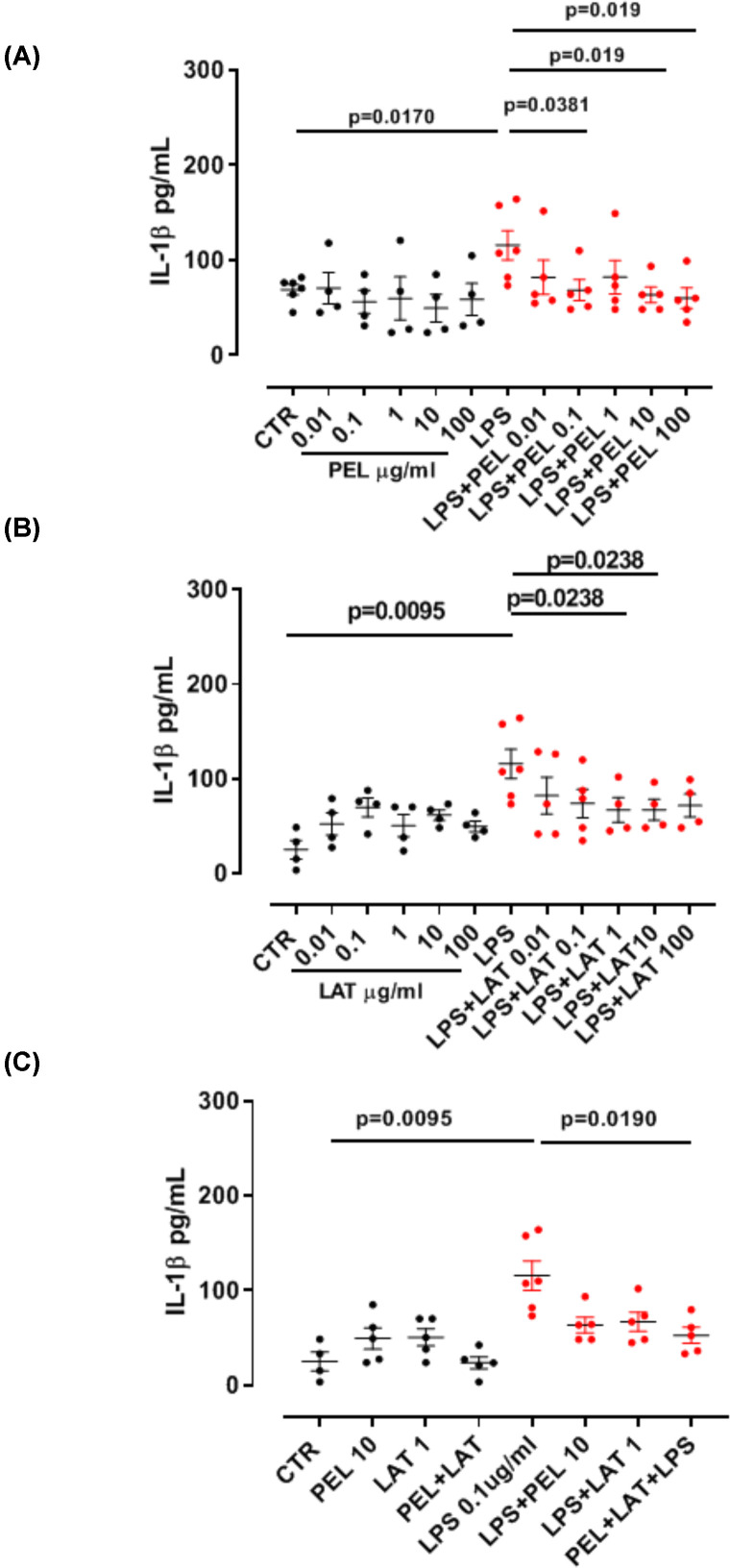
Release of proinflammatory cytokines Treatment of J774.1 murine macrophages with PEL (0.01, 0.1, 1, 10 and 100 µg/ml) (**A**), LAT (0.01, 0.1, 1, 10 and 100 µg/ml) (**B**) and PEL 10 µg/ml+LAT 1 µg/ml (**C**) in the presence or not of LPS. Black dots represent the release of IL-1β (A**–**C) after the single compound treatment, whereas the red dots represent the release of the cytokines in the presence of LPS (0.1 μg/ml). Cells were treated for 24 h. Data are represented as mean ± SEM (*n*=5). Statistically significant differences were determined by one-way ANOVA followed by Bonferroni’s multiple comparison post-test.

Because we wanted to evaluate the anti-inflammatory activity of the combination of PEL and LAT, we first analyzed the single components with the objective to identify the suboptimal concentration to use *in vitro*. J774.1 murine macrophage cells were treated for 24 h with serial concentrations of PEL (0.01, 0.1, 1, 10 and 100 μg/ml) or LAT (0.01, 0.1, 1, 10 and 100 μg/ml), in the presence or not of LPS (0.1 µg/ml), a well-known proinflammatory stimulus [12,13]. The anti-inflammatory activities were assessed by measuring the levels of IL-1β, a proinflammatory cytokine. The administration of the sole PEL (0.01, 0.1, 1, 10 and 100 μg/ml) (Figure 1A, black dots) did not alter the levels of IL-1β. In contrast, the addition of PEL on LPS-stimulated macrophages significantly reduced the levels of IL-1β at the concentration of 10–100 μg/ml (Figure 1A, red dots). Similarly, the administration of LAT alone did not alter the levels of IL-1β (Figure 1B, black dots). In contrast, the administration of LAT on LPS-treated macrophages significantly reduced the levels of IL-1β at the concentration of 1–10 μg/ml (Figure 1B, red dots).

The combination of LAT (1 µg/ml) with PEL (10 µg/ml) on LPS-treated macrophages still reduced the levels of IL-1β (Figure 1C, red dots). Because both PEL (63.65 ± 10.7 pg/ml) and LAT (67.19 ± 13.2 pg/ml) alone were able to reduce the levels of IL-1β in the presence of LPS (115.9 ± 15.4 pg/ml), we compared these levels when both PEL and LAT were added in combination onto LPS-stimulated macrophages. The levels of IL-1β were still significantly reduced when PEL+LAT (52.74 ± 8.5 pg/ml) were added onto LPS-treated macrophages compared to LPS alone (Figure 1C). In contrast, the sole addition of PEL+LAT on macrophages did not alter the levels of this cytokine (Figure 1C, black dots).

**Figure 2:**
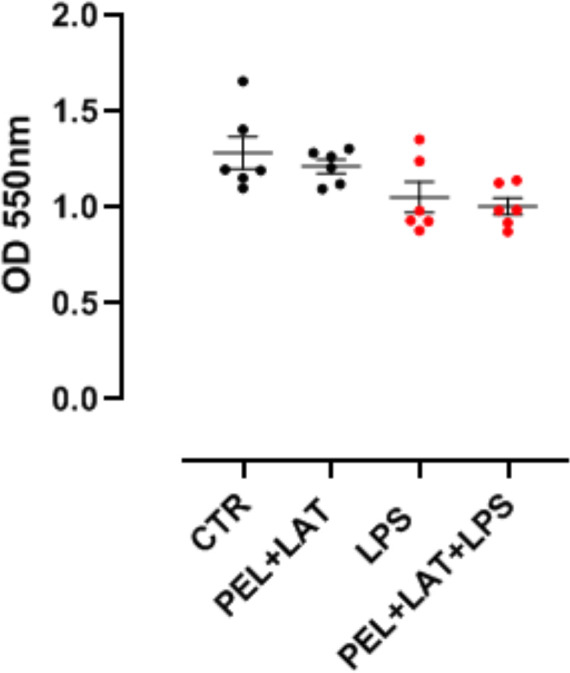
Cell viability MTT assay was performed after J774.1 murine macrophages were treated for 24 h with PEL (10 µg/ml) + LAT (1 µg/ml) and PEL 10 µg/ml + LAT 1 µg/ml + LPS 0.1 µg/ml. Data are represented as mean ± SEM. No statistically significant differences were determined according to one-way ANOVA followed by Bonferroni’s multiple comparison post-test.

In order to rule out any alteration of cell viability, MTT assay was performed and did not show any statistical reduction of cell viability either after PEL+LAT or PEL+LAT+LPS addition (Figure 2).

Taken together, these data imply that the combination of PEL and LAT had anti-inflammatory activities as shown by the levels of IL-1β.

### The combination of PEL and LAT showed anti-oxidant properties on LPS-treated macrophages

**Figure 3:**
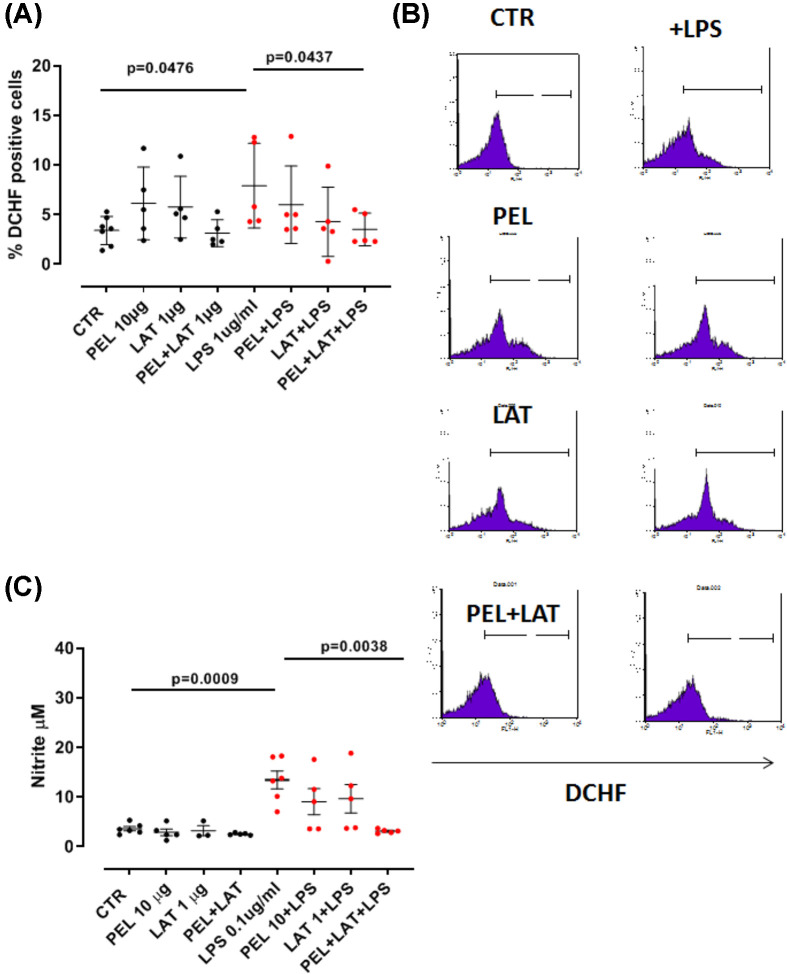
Release of reactive oxygen species and nitrite Treatment of J774.1 murine macrophages with LAT (1 µg/ml) and PEL (10 µg/ml) in the presence or not of LPS at the concentration of 1 μg/ml (A) and 0.1 μg/ml (C). The different concentration of LPS was used in accordance to the levels of oxidants (A) and nitrite (C) produced. (**A**) Black dots represent the percentage of DCHF^+^ cells after the single compound treatment, whereas the red dots represent the percentage of DCHF^+^ cells in the presence of LPS. Cells were treated for 45 min. (**B**) Representative flow histogram plots. (**C**) Black dots represent the nitrite concentration after the single compound treatment, whereas the red dots represent the nitrite concentration in the presence of LPS. Cells were treated for 24 h. Data are represented as mean ± SEM (*n*=5). Statistically significant differences were determined by one-way ANOVA followed by Bonferroni’s multiple comparison post-test.

To evaluate the antioxidant properties of the combination of PEL and LAT, J774.1 macrophagic cells were treated for 45 min with PEL (10 μg/ml) or LAT (1 μg/ml) or their combination in the presence or not of LPS (1 µg/ml). The administration of the sole LAT or PEL (Figure 3A, black dots) did not alter the percentage of DCHF^+^ cells. Instead, the administration of LAT+PEL on LPS-treated macrophages significantly reduced the percentage of DCHF^+^ cells (Figure 3A, red dots). The percentage of DCHF^+^ cells after PEL+LAT addition on LPS-treated macrophages was two-fold decreased compared with the single PEL or LAT. Indeed, the percentage of DCHF^+^ cells after PEL+LAT+LPS were 3.28 ± 1% DCHF^+^ cells versus LPS that was 6.35 ± 1.6% DCHF^+^ cells (Figure 3A,B). The sole PEL+LPS registered 6 ± 1.75% DCHF^+^ cells and LAT+LPS 4.28 ± 1.5% DCHF^+^ cells, implying that the combination was able to exert an additive antioxidant activity.

To further investigate the effect of the combination of PEL+LAT on the oxidative stress, the levels of nitrite were measured. Again, the administration of the sole PEL or LAT did not alter the levels of nitrite in cell-free supernatant (Figure 3C, black dots). In contrast, the addition of PEL+LAT robustly reduced the levels of nitrite from LPS-treated macrophages (Figure 3C, red dots). In particular, we measured the following levels of nitrite: LPS: 13.48 ± 1.8 µM, PEL+LPS: 9.2 ± 3.4 µM, LAT+LPS: 9.69 ± 3.7 µM, PEL+LAT+LPS: 3.13 ± 0.2 µM. The administration of PEL+LAT showed a potentiated pharmacological activity compared with the single compound.

Taken altogether, these data suggest that the combination of PEL+LAT exerted a potentiated antioxidant activity on LPS-treated macrophages.

### PEL+LAT combination had antimicrobial activities

It is already known that PEL and LAT have antimicrobial activities [3,14]. However, the main goal of the present study was to understand whether the combination of both could prove as antimicrobial drug. Therefore, we used Gram positive bacterium, *S. aureus* (0.5 × 10^6^ CFU/ml), and Gram negative bacterium, *E. coli* (0.5 × 10^6^ CFU/ml) cultures that were treated with LAT at the MIC of 1 μg/ml or PEL at the MIC of 10 μg/ml. Bacterial growth was evaluated 1, 2, 3, 4, 5, 6, 7 and 20 h after treatment.

We already proved that the administration of PEL showed bacteriostatic activities on both *S. aureus* and *E. coli* growth at the MIC of 10 µg/ml, starting from 5 and 6 h, respectively, after treatment compared to the control group [5].

**Figure 4:**
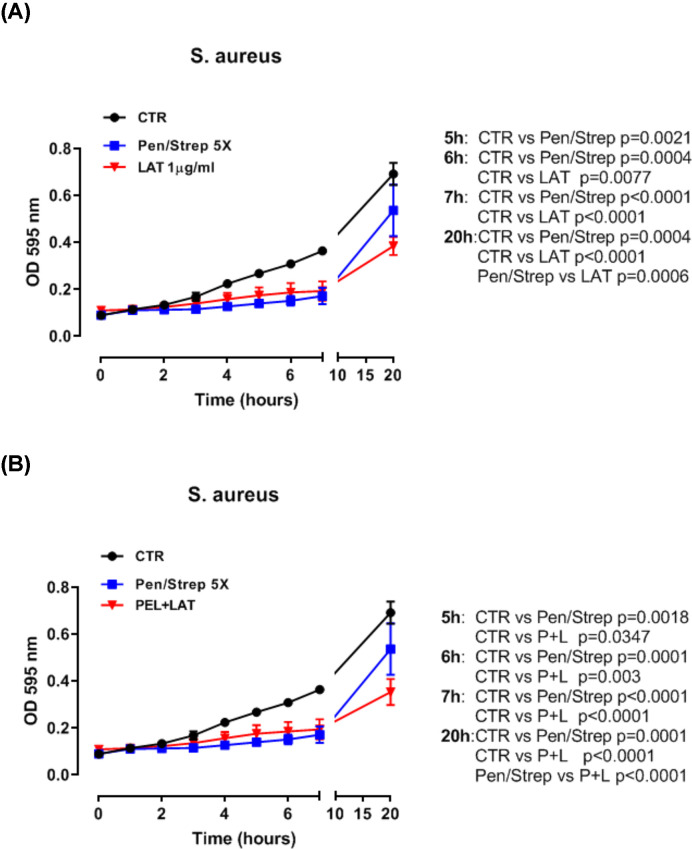
*S. aureus* growth Treatment of *S. aureus* (0.5 × 10^6^ CFU/ml) culture with LAT (1 µg/ml) (**A**) and PEL+LAT (**B**). The black line represents the control of the bacteria growth without antibiotic. The blue line represents bacteria growth with penicillin/streptomycin as antibiotic, and the red line represents the bacteria growth with LAT alone (A) or in combination with PEL (B). The bacteria growth was analyzed from 0 up to 20 h. Data are represented as mean ± SEM range (*n*=9). Statistically significant differences were determined by two-way ANOVA followed by Tukey’s multiple comparison post-test.

The administration of LAT (1 µg/ml) significantly reduced the growth of *S. aureus* starting from 6 h post treatment compared with the control group (Figure 4A, red line versus black line). This effect was comparable to the activity of the mixture penicillin/streptomycin (Pen/Strep) (Figure 4A, blue line versus red line). However, LAT showed higher antimicrobial activity in terms of *S. aureus* growth compared with Pen/Strep mixture at 20 h (Figure 4A, red line versus blue line). The combination of PEL+LAT showed an earlier activity (5 h) (Figure 4B) than LAT alone (Figure 4A) in that the growth of *S. aureus* was reduced at 5 h compared with 6 h when LAT was added alone.

**Figure 5:**
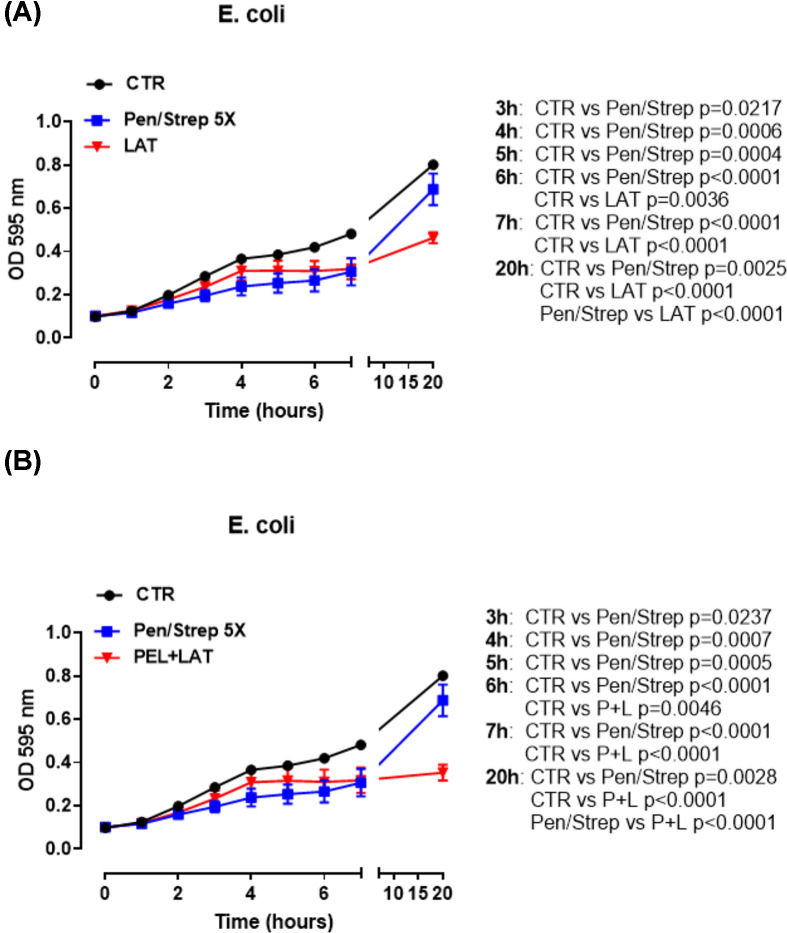
*E. coli* growth Treatment of *E. coli* (0.5 × 10^6^ CFU/ml) culture with LAT (1 µg/ml) (**A**) and PEL+LAT (**B**). The black line represents the control of the bacteria growth without antibiotic. The blue line represents bacteria growth with penicillin/streptomycin as antibiotic, and the red line represents the bacteria growth with the combination. The bacteria growth was analyzed from 0 up to 20 h. Data are represented as mean ± SEM range (*n*=9). Statistically significant differences were determined by two-way ANOVA followed by Tukey’s multiple comparison post-test.

Similarly, the administration of LAT significantly reduced the growth of *E. coli* culture at 6 h compared with the control group (Figure 5A, red line versus black line). The combination of PEL+LAT significantly reduce the growth of the *E. coli* culture at 6 h post-treatment (Figure 5B). Interestingly, this effect was more pronounced than that observed for Pen/Strep at 20 h (Figure 5B, blue line versus red line).

## Discussion

The main goal of the present study was to evaluate whether the combination of PEL and LAT could exert additive/synergistic pharmacological activities as anti-inflammatory, antioxidant and antimicrobial agents compared with the single components. Similarly to what reported in literature, we found that PEL and LAT used alone were able to reduce LPS-induced proinflammatory IL-1β, as well as reduce reactive oxygen species (ROS), nitrite and bacteria growth. More importantly, the combination of PEL with LAT showed an additive pharmacological activity in terms of antioxidant and antimicrobial activities.

IL-1β is widely described as a proinflammatory cytokine, released after LPS stimulation [15,16]. Our data demonstrate that the combination of PEL+LAT significantly reduced the levels of IL-1β after LPS stimulation. This effect could prompt PELIRGOSTIM as an innovative and hitherto unknown combination, able to attenuate inflammation-related pathways. IL-1β is a critical regulator of the inflammatory response that, according to the activation of the inflammasome complex, its release can promote leukocyte migration with ensuing tissue damage, can promote T-cell survival, can contribute to the polarization of Th1, Th2 and Th17 differentiation. In addition, IL-1β induces IL-6 and/or TNF-α release, participating to the recruitment of other immune cells which phenotype can be endeavored according to the microenvironment [16]. However, our data have the limitation to prove the sole activity of the combination PEL+LAT to reduce IL-1β release, but future studies will be performed by using *in vivo* models to prove its anti-inflammatory activity. Nevertheless, we believe that the capability of PEL+LAT to reduce the release of IL-1β from LPS-treated macrophages, associated with the reduced oxidant activity, can be of beneficial in inflammatory-based disorders.

Indeed, another important characteristic of PEL+LAT combination is the antioxidant activity, which could be of potential relevance in oxidative stress-based pathologies, such as chronic obstructive pulmonary disease (COPD) [12,17,18]. In particular, we observed that PEL+LAT exerted a potentiated antioxidant activity on LPS-treated macrophages. Indeed, although the reduction of ROS (DCHF^+^ cells) and of nitrite by the single PEL or LAT, the combination PELIRGOSTIM was able to reduce the levels of ROS in a 2-fold manner in the presence of LPS, as well as to reduce nitrite in a 4-fold manner. This effect is of great clinical relevance especially in pathologies, during which the oxidative stress plays a pivotal role. Therefore, the identification of beneficial nutraceuticals, as in the case of PEL and LAT, could be important to prevent the establishment of an oxidative status that leads cells to damage ensuing an inflammatory pattern that can be at the basis of the pathology.

Another important issue that deserves to be highlighted in this manuscript is that the combination of PEL and LAT exerted higher antimicrobial activity than LAT alone, well-known to be important during breast feeding [19]. PEL+LAT were able to statistically reduce *S. aureus* and *E. coli* growth, effect that was longer than that observed for penicillin/streptomycin. The administration of the single compounds still reduced bacteria growth; however, this effect was observed at 6 h rather than at 5 h. Moreover, this effect was not due to antioxidant activity of the single compounds as already reported [4,20]. Indeed, the sole compounds were not able to exert antioxidant activities, rather, they showed bacteriostatic activity although at later time points. It has already been reported that LAT has strong antimicrobial activities against Gram positive and Gram negative bacteria as well as fungi, yeasts, viruses and protozoa [14]. The antimicrobial activity of LAT is generally associated to two mechanisms: the first is based on iron absorption in the site of infection, leading to the deprivation of nutrients to microbes; the second one is the direct contact of LAT with pathogens which interferes with microbial physiology [21]. In the latter case, the presence of positive amino acids in LAT chemical structure allows it to interact with negatively charged molecules on some bacterial, viral, fungal and parasite surfaces, causing cell lysis [21]. On the other hand, we already proved that PEL alone can exert antimicrobial activities [5]. Here, the combination of PEL and LAT led to an additive bacteriostatic activity, especially in the case of *S. aureus*. In addition, due to the recent emergency and spread of multidrug resistant pathogens, the combination of PEL and LAT could prove of innovative potentiality for the actual antimicrobial therapy.

## Conclusion

In conclusion, although the limitation of an *in vitro* study, these data suggest that the combination PEL+LAT (PELIRGOSTIM) can reduce the release of proinflammatory cytokines, oxidants and bacteria growth, most likely preventing leukocyte chemiotaxis with a reduced inflammatory pattern. Therefore, our data pave the way for an innovative therapeutic approach for inflammation-, oxidative stress- and microbial-related disorders.

## Abbreviations

COPD: chronic obstructive pulmonary disease
DCHF-DA: 2′,7′-dichlorodihydrofluorescein diacetate
DMEM: Dulbecco’s Modified Eagle Medium
LAT: lactoferrin
MIC: minimum inhibitory concentration
MTT: 3-(4,5-dimethylthiazol-2-yl)-2,5-diphenyltetrazolium bromide
ROS: reactive oxygen species
